# Reproductive and lactational responses of multiparous dairy cattle to short-term postpartum chromium supplementation during the summer months

**DOI:** 10.3168/jdsc.2022-0287

**Published:** 2022-12-22

**Authors:** Dallas R. Soffa, Jacob W. Stewart, Alicia G. Arneson, Nicholas W. Dias, Vitor R.G. Mercadante, Robert P. Rhoads, Michelle L. Rhoads

**Affiliations:** Department of Animal and Poultry Sciences, Virginia Tech, Blacksburg 24061

## Abstract

The objective of this work was to evaluate the potential benefits of short-duration, high-dose chromium (Cr) supplementation in early postpartum dairy cows during the summer months. Multiparous, early-lactation cows (20.95 ± 0.21 d in milk) were assigned to 1 of 2 treatment groups: (1) control diet (Con; n = 10) or (2) control diet + Cr propionate (CrPro; 12 mg/head per day Cr; n = 12). Measurements of ovarian structures, respiration rates (RR), rectal temperatures (RT), and blood glucose concentrations were recorded every 3 d. Blood was also collected for analysis of plasma progesterone concentrations. Every 6 d, in conjunction with ultrasonography, endometrial cytology samples were collected via cytobrush from each cow to determine the incidences of subclinical endometritis, as determined by polymorphonuclear leukocyte (PMNL)%. No differences were detected in RR, RT, blood glucose, feed intake, milk yield, or change in body weight. The supplementation did, however, improve some reproductive parameters. At cytology sample 6, the PMNL% increased in Con cows, and was greater than the PMNL% in the CrPro group. Chromium consumption did not affect the number or size of most follicles, with the exception being the 6 to 9 mm category where the CrPro group had a greater average diameter and tended to have greater numbers of follicles in this category. While corpus luteum numbers and size did not differ between treatments, the ratio of progesterone to average corpus luteum volume was greater in the CrPro group compared with the Con group. The results from this study indicate that, whereas the short-term, high-dose supplementation strategy did not affect feed intake or milk yield, this Cr supplementation strategy could benefit reproductive performance during periods of stress.

## Highlights

• Multiparous Holstein dairy cows (n = 22) in early lactation were enrolled during seasonal heat stress.
• Cows either received or did not receive a chromium (Cr) feed supplement during the treatment period.
• Chromium consumption did not affect milk yield, feed intake, blood glucose, or rectal temperatures.
• Chromium supplementation improved endometrial polymorphonuclear leukocyte percent.
• Chromium intake increased the size and number of small follicles and progesterone ratio.

## Graphical Abstract

**Figure:**
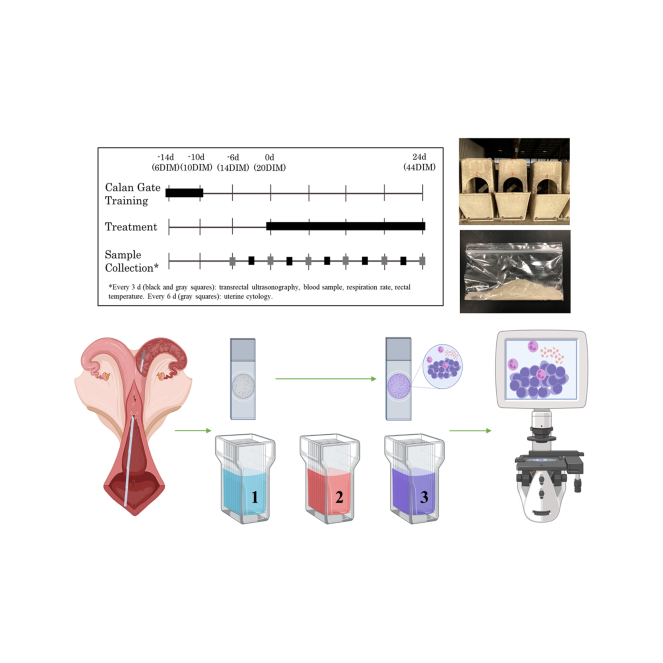
**Summary:** Twenty-two multiparous, lactating Holstein cows were enrolled in the experiment, spanning the summer months of June through September. Cows were housed in freestalls where they were individually fed using a Calan gate system. Cows were randomly assigned to the control (Con; no supplement; n = 10) or chromium propionate (CrPro; 12 mg/head per day chromium for 24 d; n = 12) treatment groups. Every 3 days, ovarian structures were counted and measured and blood samples, respiration rates, and rectal temperatures were collected. Every 6 days, a uterine swab was obtained for cytological evaluation to determine the incidences of subclinical endometritis, as determined by polymorphonuclear leukocyte percentage. Chromium supplementation benefited small ovarian follicles, polymorphonuclear leukocyte percentage, and the ratio of plasma progesterone to average corpus luteum volume.

During periods of stress, such as summer heat exposure and the early postpartum period, individual physiological responses can reduce productivity, thereby decreasing economic profit for dairy producers. The severity of the responses to stress can be diminished with chromium (Cr) supplementation (Mirzaei et al., 2011). Chromium is an essential trace mineral (Soltan, 2010) known for its ability to enhance insulin-mediated glucose uptake in dairy cattle, which, in practice, often results in an increase in milk production. The improvement in energetic status following Cr consumption can also positively affect reproductive performance, immune function, and responses to stressors (Burton et al., 1996; Mirzaei et al., 2011; Negrón-Perez et al., 2019).

The early postpartum period is a phase of production during which dairy cows are particularly susceptible to a variety of stress-related disorders. The transition in homeorhetic priority from maintaining pregnancy to producing substantial quantities of milk is a major physiological shift associated with a plenitude of maladies (Esposito et al., 2014). Dairy cows calving in the late spring or summer have the added physiological challenges associated with making this transition during heat stress (Negrón-Perez et al., 2019). The physiological responses to elevated ambient temperatures are a major concern for the dairy industry, as they are associated with approximately $1.5 billion in economic losses each year for dairy operations in the United States. These losses result from combined effects of heat stress on milk yield, reproductive performance, disease incidence, and culling rates (St-Pierre et al., 2003).

The ability of Cr to improve postpartum energetic status and the physiological responses to stressors makes it an ideal candidate for supplementation to heat-stressed early-lactation dairy cows. Previous research, however, has typically relied on lengthy supplementation periods during which Cr treatment is initiated before calving (Burton et al., 1996; Soltan, 2010; Yasui et al., 2014). We hypothesized that short-term, high-dose Cr supplementation initiated after calving (rather than during the dry period) would improve production parameters in early postpartum dairy cows during the summer months.

All animal procedures were approved by the Virginia Tech Institutional Animal Care and Use Committee (IACUC). Twenty-two multiparous (2.86 ± 0.34 lactations), lactating Holstein cows (658.29 ± 13.61 kg of BW) were housed in one of two 12-stall pens within a freestall barn for the duration of the experiment, spanning the months of June through September. Milk production was recorded twice daily (0000 and 1200 h), and BW were collected after the second milking each day. Cows were individually fed once daily (1100 to 1400 h) using a Calan gate system (American Calan Inc.).

Throughout the experiment, all cows were fed the same base TMR formulated to meet or exceed the nutrient requirements of early lactation (National Academies of Sciences and Medicine, 2021). Cows were fed for ad libitum intake and feed refusals were collected and weighed daily. On d 0 (20.95 ± 0.21 DIM), each cow was randomly assigned to (and began) either the control (**Con**; n = 10) or Cr propionate (**CrPro**; n = 12) treatment group, based upon parity and average milk production over the 3 previous d. Cows assigned to the CrPro group, received 30 g of CrPro daily for 24 d (12 mg of Cr per head per day; KemTRACE chromium propionate; Kemin Industries Inc.). The supplement was administered by individually top-dressing and then hand-mixing into the top one-third of the TMR ration.

In the morning of every third day from −6 to 24 d, transrectal ultrasonography was conducted (IBEX PRO portable ultrasound with a L7HDi linear transducer; E.I. Medical Imaging). Follicles were counted (all follicles), measured (all follicles > 5 mm), and categorized by size according to Lucy et al. (1991). Likewise, the number and average diameter of all luteal structures were evaluated. The volume of the corpus luteum (**CL**) was calculated from the measurements of diameter (Ricci et al., 2017). Fluid-filled cavities within the CL were noted, but because the cavities were sometimes not spherical (as is frequently observed during routine ultrasound) and are not associated with reductions in circulating progesterone (Kito et al., 1986; Jaśkowski et al., 2022), they were not included in the calculation of CL volume (Assey et al., 1993).

Rectal temperatures (**RT**), respiration rates (**RR**), and blood samples (coccygeal venipuncture; sodium heparin vacutainers; Becton, Dickinson and Company) were also collected every third day, before transrectal ultrasonography. Blood glucose concentrations were immediately determined using a handheld glucometer (Contour Next EZ; Ascensia Diabetes Care US Inc.). The remainder of each blood sample was placed on ice until plasma was collected following centrifugation at 3,000 × *g* and 4°C for 15 min.

Every other procedure day (every 6 d), starting at −6 d, uterine cytology samples were also collected before transrectal ultrasonography as described in Kasimanickam et al. (2004). Samples were collected using a cytology brush (Cytobrush Plus GT; CooperSurgical Inc.) attached to the plunger of an AI rod that had been sterilized in a chlorhexidine solution. After collection, swabs were immediately rolled onto a glass slide and allowed to air dry. Each slide was sprayed with a fixative (CytoPrep Fixative; Electron Microscopy Sciences) and then stored until staining.

After all slides were collected from all cows, they were stained with a modified Giemsa stain (Differential Quik Stain Kit; Electron Microscopy Sciences) with slight modifications to the manufacturer's recommended procedures. Briefly, each slide was first dipped for 10 s in a fixative solution, followed by 1 min in an eosin Y-based solution. The slides were then dipped 2× in the fixative solution, before being dipped 2× in a methylene blue trihydrate-based solution, then rinsed in distilled water and allowed to air dry. The slides were then dipped 10× each in 100% ethanol and xylene, before 2 drops of a toluene-based histological mounting medium (Permount Mounting Medium; Electron Microscopy Sciences) were placed on the sample for coverslip mounting. Coverslips were allowed to dry overnight. Two hundred cells were randomly counted by 2 observers blinded to the treatments, and the number of polymorphonuclear leukocytes (**PMNL**) within the 200 cells averaged to determine the PMNL%. Red blood cell (**RBC**) contamination was also assessed (scores averaged between 2 observers) as described in Pascottini et al. (2015).

Progesterone concentrations in plasma samples were determined using an Immulite 2000 Progesterone solid-phase, competitive chemiluminescent enzyme immunoassay (Immulite 2000 XPi platform; Siemens Medical Solutions Inc., USA; Podico et al., 2020). All samples were run in a single assay with an intraassay coefficient of variation of 1.99%.

Climatic data were obtained (National Oceanic and Atmospheric Administration) and included dry bulb temperature (**T_dry bulb_**) and dew point temperature (**T_dew point_**) observed at 15-min intervals for the full duration of the study. Temperature-humidity index (**THI**) values were calculated from hourly mean T_dry bulb_ and T_dew point_ as described in Brown et al. (2016).

One cow was removed from the experiment for reasons unrelated to the treatment. Data were analyzed using the MIXED procedure of SAS (SAS Institute Inc.). Independent variables were treatment (Con or CrPro), day of experiment, and their interaction. Cow was included in the model as the subject from which repeated measurements were taken. When day of experiment or the interaction between treatment and day of experiment were not significant, they were removed from the model. For analysis of PMNL%, RBC score was included as a covariate. The ratio of plasma progesterone to CL volume was calculated and analyzed using 2 separate approaches for each cow on each sample day: (1) plasma progesterone concentration/average CL volume, and (2) plasma progesterone/total CL volume. For each analysis, 8 covariance structures were tested and the most appropriate was selected based upon Akaike's information criterion, Akaike's information criterion with correction, and Bayesian information criterion values. Pairwise comparisons were conducted using Tukey's procedure. The probabilities of the presence of a CL or subclinical endometritis (**SCE**) at the time of sample collection was analyzed using the FREQ procedure of SAS as well as mixed-effect logistic regression. Results are reported as least squares means ± standard errors of the means. Statistical significance was declared at *P* ≤ 0.05 and tendencies at 0.05 ≤ *P* ≤ 0.10.

Previous studies have reported changes in milk production when Cr supplementation levels were as low as 6 mg per head per day and initiated during the dry period (Soltan, 2010). In contrast, the current study was designed to determine whether initiating supplementation approximately 3 wk postcalving (thus, short-term) could benefit early-lactation dairy cows experiencing thermal stress as they approach the end of the voluntary waiting period. Since the duration of supplementation was short in comparison to most previous experiments, the amount of Cr administered in this study was greater than most previous experiments (thus, high-dose), yet still within the manufacturer's recommended dose range (Soltan, 2010; Kafilzadeh et al., 2012; Yasui et al., 2014).

Pre-treatment feed intake, milk yield, and the feed intake to milk yield ratio were all similar between cows that were ultimately assigned to Con and CrPro treatments. Initial BW did tend to differ (642.1 ± 19.4 kg and 688.3 ± 17.7 kg of BW for Con and CrPro, respectively; *P* = 0.09) and this tendency was maintained throughout the experiment (632.1 ± 19.2 kg and 675.7 ± 17.5 kg of BW during treatment for Con and CrPro, respectively; *P* = 0.11). As such, the change in BW (d 0 BW to d 24 BW) was also evaluated and did not differ between treatment groups (Table 1). Likewise, feed intake, milk yield, and the feed intake to milk yield ratio were unaffected by treatment (Table 1). While these results differ from many previous experiments (Soltan, 2010; Mirzaei et al., 2011), they are consistent with others (Yang et al., 1996; Yasui et al., 2014), indicating that Cr-specific effects on feed intake and milk yield can vary with supplementation strategy and other undetermined variables.

**Table 1:** Mean physiological parameters (LSM ± SEM) of early-lactation dairy cows that did (CrPro) or did not (Con) receive chromium supplementation^1^

Parameter^2^	Con	CrPro	*P*-value
Feed intake (kg)	44.66 ± 1.78	42.33 ± 1.63	0.35
Milk yield (kg)	57.75 ± 2.28	57.12 ± 2.09	0.84
Feed intake:milk yield (kg)	0.79 ± 0.03	0.75 ± 0.03	0.39
Change in BW (kg)	−1.18 ± 4.56	−0.63 ± 4.56	0.93
Respiration rate (bpm)	66.97 ± 1.40	68.19 ± 1.31	0.52
Rectal temperature (C°)	39.20 ± 0.04	39.16 ± 0.04	0.46
Blood glucose (mg/dL)	58.96 ± 0.52	58.41 ± 0.49	0.44
Number of CL	1.27 ± 0.08	1.38 ± 0.08	0.34
CL diameter (mm)	15.37 ± 0.72	15.65 ± 0.67	0.78
Number of follicles <5 mm	5.18 ± 0.47	5.56 ± 0.45	0.56
Number of follicles 6–9 mm	1.88 ± 0.17	2.26 ± 0.16	0.09
Diameter of 6–9 mm follicles (mm)	6.95 ± 0.09	7.21 ± 0.08	0.04
Number of follicles 10–15 mm	0.87 ± 0.09	0.95 ± 0.08	0.52
Diameter of 10–15 mm follicles (mm)	11.85 ± 0.17	11.54 ± 0.16	0.18
Number of follicles >15 mm	0.35 ± 0.06	0.24 ± 0.06	0.17
Diameter of 15 mm follicles (mm)	16.46 ± 0.42	16.61 ± 0.43	0.79
Incidence of SCE (%)	17.50	13.72	0.54

1 Control (Con; n = 10); chromium supplementation (CrPro; 12 mg/head per day Cr; n = 12).

2 CL = corpus luteum; SCE = subclinical endometritis.

Calculated THI values indicated that cows enrolled in this study were exposed to stressful thermal conditions (THI levels of 68 or greater; Zimbelman et al., 2009) for 14 h per day (data not shown). Because of scheduling constraints, RR and RT were only collected in the mornings (between 0600 and 1100 h) in conjunction with transrectal ultrasound. Even though these measurements were collected in the morning hours following the overnight THI nadir, they were greater than would be expected during thermoneutral conditions and consistent with morning measurements of lactating dairy cattle exposed to controlled heat stress (Table 1; Rhoads et al., 2009). Some previous studies have found that when livestock were subjected to heat stress, Cr supplementation reduced RR and RT, particularly during the hottest hours of the day (Liu et al., 2017; Hung et al., 2021). Dietary supplementation of Cr, however, did not improve RR or RT in the current experiment. The lack of difference in either parameter during Cr supplementation is likely the result of the time of day at which RR and RT measurements were conducted (not during maximum THI). Other heat-stress studies have also failed to detect an improvement in RR or RT during Cr supplementation (Mirzaei et al., 2011; Mayorga et al., 2019).

Chromium is a potent regulator of insulin-mediated glucose uptake, which typically causes circulating glucose concentrations to be lesser in Cr-supplemented animals (Mowat et al., 1993; Depew et al., 1998; Stahlhut et al., 2006). This is not always the case, however, particularly for those studies involving heat stress (Soltan, 2010; Mirzaei et al., 2011; Mayorga et al., 2019). Likewise, blood glucose concentrations in the current experiment did not differ between treatment groups (Table 1). Previous work from our laboratory and others suggests that insulin-stimulated glucose uptake is already maximized in heat-stressed dairy cattle (Rhoads et al., 2009; Xie et al., 2016; Stewart et al., 2022). Consequently, it is likely that Cr is unable to further increase glucose uptake under these conditions.

Studies have shown that both heat stress and energetic status can affect circulating progesterone concentrations and the length of the luteal phase (De Rensis and Scaramuzzi, 2003). If disruptions are severe enough, estrous cyclicity or the establishment of pregnancy (or both) will be detrimentally affected (Bishop et al., 2022). In the current study, neither the number of CL nor their average diameter were affected by Cr supplementation (Table 1). Maximum plasma progesterone concentrations could not be determined because blood samples were not collected frequently enough (samples were collected every 3 d). Instead, the ratios of progesterone concentration to CL volume were determined using 2 separate approaches. The analysis utilizing total CL volume yielded no differences between the Con and CrPro groups (0.54 ± 0.06 and 0.59 ± 0.06 ng/cm^3^, respectively; *P* = 0.56). Interestingly, even though there were no differences in CL number between treatments, the analysis using the average CL volume as the denominator demonstrated that the cows consuming the CrPro produced more progesterone per average unit of CL volume than the Con group (Figure 1). Because the size of the CL is roughly related to its age (Kayacik, 2005; Ricci et al., 2017), this analysis suggests that if blood samples had been collected more frequently (i.e., if peak progesterone values could have been determined), the maximum plasma progesterone concentrations may have been greater in the CrPro treatment group than in Con. Additional research is needed to confirm this speculation. Ultimately, however, greater circulating progesterone concentrations could increase the likelihood of pregnancy recognition and subsequent establishment of pregnancy.

**Figure 1:**
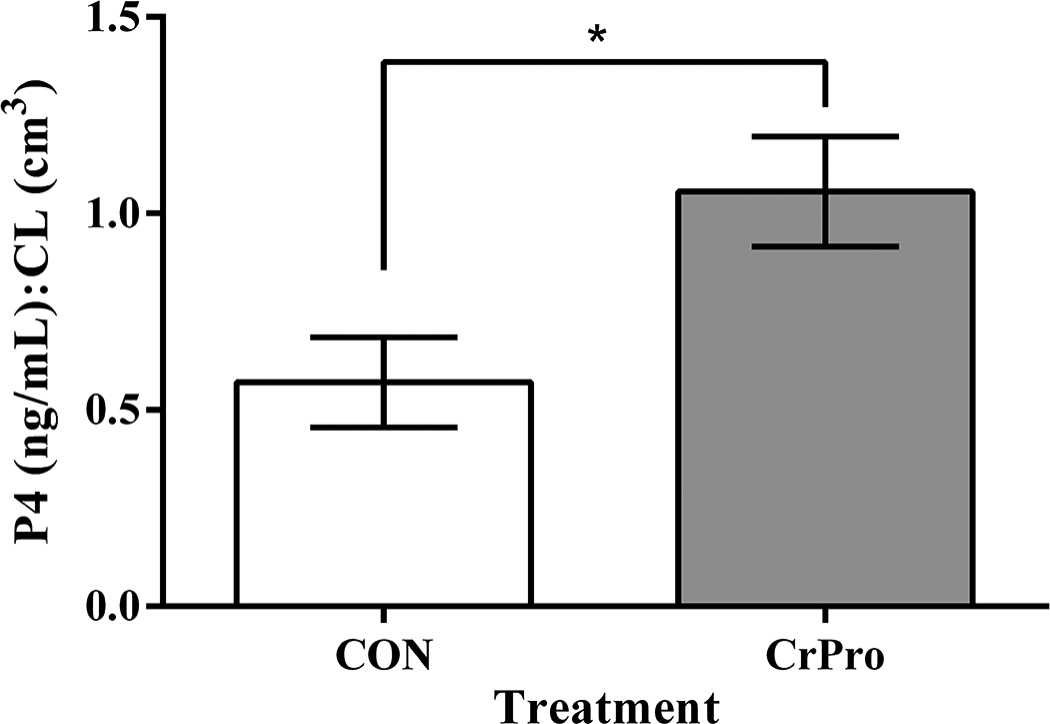
Mean (LSM ± SEM) ratios of progesterone (P_4_; ng/mL) to average corpus luteum (CL) volume (cm^3^) differed between the control (Con) and chromium propionate (CrPro) treatment groups. *P = 0.03.

Follicle development during the early postpartum period is linked with subsequent fertility in lactating dairy cows (Furukawa et al., 2020; Sood et al., 2022) and can be detrimentally affected by heat stress (De Rensis and Scaramuzzi, 2003). Chromium supplementation in the current study affected 2 follicular measurements (Table 1). The CrPro treatment increased the average diameter of the follicles in the small category and tended to increase the number of small follicles (6–9 mm in size). This increase in number and size of the recruited follicles was not sustained, however, as the numbers and diameters of larger follicle categories did not differ between treatments (Table 1).

Heat stress and the early postpartum period are both associated with an increased risk for metritis in dairy cattle. The incidence of endometritis within some dairy herds exceeds 50% (Cheong et al., 2011). Endometritis is problematic for several reasons, but is perhaps best known for its effect on postpartum fertility, as indicated by reduced first service pregnancy rates, increased services per conception, and greater median days open (Sheldon et al., 2020). In this study, individual cows did occasionally meet or exceed the thresholds for SCE diagnosis (Gilbert et al., 2005). At no point, however, did mean PMNL% for either treatment group exceed the threshold for SCE diagnosis (Figure 2). The incidences of SCE also did not differ between treatments (Table 1).

**Figure 2:**
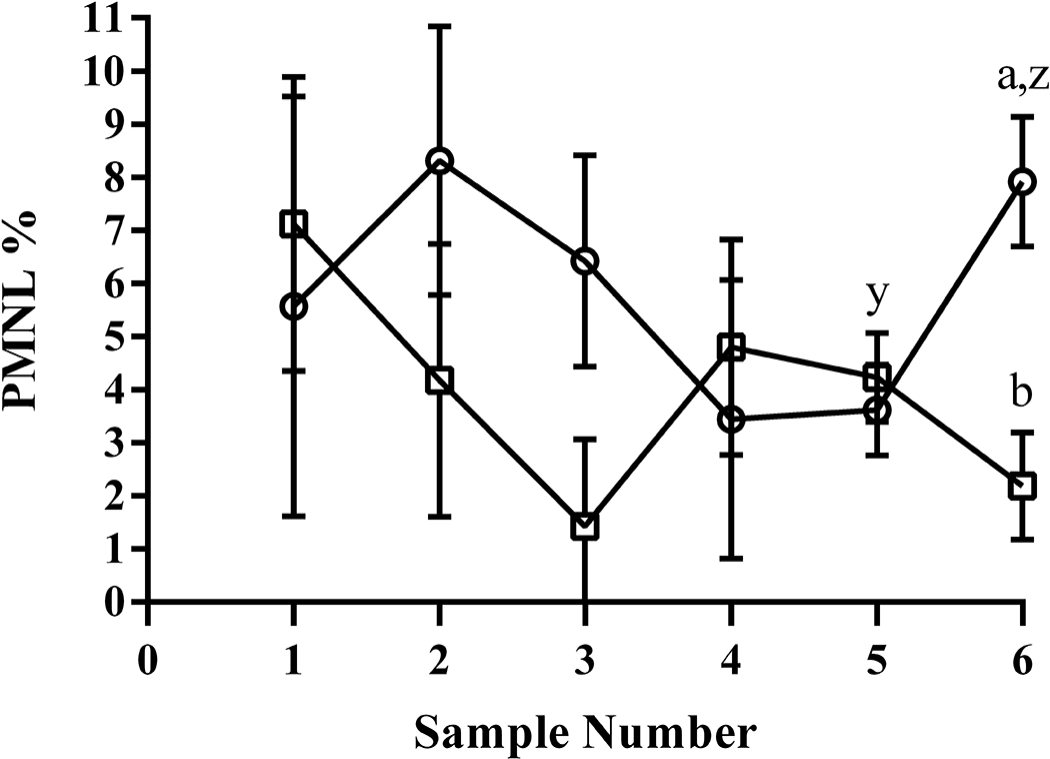
Mean (LSM ± SEM) percentage of polymorphonuclear leukocytes (PMNL) counted in the endometrial cytology samples differed between control (Con; circles) and chromium propionate (CrPro; squares) treatments (*P* = 0.01). (a, b) denotes significance (*P* = 0.01) between treatment groups; (y, z) denotes significance (*P* = 0.02) within the Con group.

Within the Con group, the PMNL% increased between samples 5 and 6 (*P* = 0.02; Figure 2). This increase in PMNL% at the end of the sampling period could be indicative of a resurgence in endometrial inflammation. Although we cannot rule out the possibility that this was the result of repeated endometrial swabs (the swabs may partly explain the Con group PMNL%), it is important to note that the cows in the CrPro treatment were subjected to the same number (and timing) of procedures and did not exhibit an increase in PMNL%. Thus, the uterine PMNL% was greater in the Con group compared with the CrPro group at sample time point 6 (*P* = 0.01; Figure 2). The difference in PMNL% between Con and CrPro-treated cows is supported by previous work where the incidence of SCE at 40 to 60 d postpartum was reduced by CrPro supplementation (Yasui et al., 2014). Together, these results indicate that Cr supplementation may aid in improving the uterine environment in postpartum dairy cows. Any strategy capable of improving the uterine environment also has the potential to increase fertility. In the current study, however, there were not enough cows enrolled to evaluate pregnancy rates following supplementation.

Overall, the short-duration, high-dose CrPro supplementation strategy implemented in this study did not affect blood glucose concentrations, RR, RT, feed intake, and milk yield. Although these variables were not affected by CrPro supplementation, it did improve reproductive parameters, including the ratio of progesterone to average CL volume, PMNL%, and characteristics of small ovarian follicles. These results indicate that CrPro supplementation could act to improve subsequent pregnancy recognition, lower SCE rates, and ultimately improve fertility. Additional research following cows through AI and pregnancy detection is needed to confirm these assertions. Ultimately, the short-duration, high-dose CrPro supplementation strategy benefited aspects of reproductive performance of early-lactation dairy cattle during the summer months.
